# Assessment of Peripapillary Retinal Nerve Fibre Layer Thickness, Optic Nerve Head Rim Area, Anterior Chamber Parameters, and Axial Length in Myopic Eyes

**DOI:** 10.7759/cureus.94271

**Published:** 2025-10-10

**Authors:** Majid A. Moafa, Samah Samy Albahrawy, Muhammed S Alluwimi, Saif Alrasheed, Abdelaziz M Elmadina, Raghda F Mutwaly, Ahmed Fouad Abdelhamid, Eyad Hamzah Albarnawi, Waleed M Alghamdi, Yousef H Aldebasi

**Affiliations:** 1 Department of Optometry, College of Applied Medical Sciences, Qassim University, Buraydah, SAU; 2 Department of Ophthalmology, The Royal Commission Medical Center, Medina, SAU

**Keywords:** anterior chamber depth, clinical feature, myopia, retina, young adult

## Abstract

Background: Myopic eyes commonly show structural changes, including optic nerve head (ONH) and altered anterior chamber parameters.

Aim: The study aimed to investigate the associations of peripapillary retinal nerve fibre layer (RNFL) thickness, optic disc rim area, anterior chamber parameters, and axial length in myopic eyes.

Methods: This cross-sectional study was conducted at the Royal Commission Medical Centre, Yanbu, Saudi Arabia, between February and May 2025 and included 152 myopic eyes. Refraction was measured using an autorefractometer. Swept-source optical coherence tomography (SS-OCT) scans (optic disc cube 200 × 200) were used to assess peripapillary RNFL thickness and optic disc rim area. Corneal and anterior chamber parameters, along with axial length, were measured using Pentacam AXL (OCULUS Optikgeräte GmbH, Wetzlar, Germany). Data analysis was conducted to assess the correlation between myopia severity and structural ocular changes.

Results: Myopic eyes (mean spherical equivalent: -2.02 ± 1.34 D) showed a significant inverse correlation with both central corneal thickness (r = -0.193, P < 0.05) and corneal thickness at the thinnest point (r = -0.225, P < 0.05). Anterior chamber volume (r = 0.266, P < 0.001) and anterior chamber depth (r = 0.259, P < 0.001) showed significant positive correlations with myopia, while the anterior chamber angle showed no significant association (P > 0.05). Axial length was strongly correlated with myopia severity (r = 0.545, P < 0.001). Inverse correlations were observed between myopia and both peripapillary RNFL thickness (r = -0.100, P > 0.05) and ONH rim area (r = -0.134, P > 0.05). A statistically significant inverse correlation was found between peripapillary RNFL thickness and axial length (r = -0.163, P < 0.05), as well as between ONH rim area and axial length (r = -0.167, P < 0.05).

Conclusion: The study revealed a significant positive correlation between myopia and both anterior chamber volume and anterior chamber depth, while the anterior chamber angle remained unaffected. The increase in myopia severity was correlated with thinning of the peripapillary RNFL and a reduction in ONH rim area. These findings underscore the importance of assessing axial elongation and structural changes in myopic eyes, particularly in the context of ocular diseases such as glaucoma, myopic macular degeneration, and retinal detachment.

## Introduction

Myopia is a common refractive error and is usually caused by increased axial length, leading to light focusing in front of the retina. Its global prevalence is rising rapidly, with projections suggesting that, by 2050, nearly 50% of the world population will be affected, and about 10% will develop high myopia [1]. Recent studies [2,3] have shown a significant increase in myopia, reaching epidemic proportions, particularly in urbanised and industrialised areas. The rising prevalence of myopia is particularly concerning due to its strong association with numerous vision-threatening ocular complications. High myopia is considered a risk factor for conditions such as retinal detachment, myopic maculopathy, posterior staphyloma, choroidal neovascularisation, and glaucomatous optic neuropathy [4, 5]. These complications have a profound impact on quality of life and place a significant burden on healthcare systems, particularly in regions experiencing rising myopia rates, such as the Middle East [6,7]. Moreover, recent estimates in Saudi Arabia indicate that the prevalence of myopia is approximately 17% [8].

Despite the growing recognition of the epidemiological and clinical impact of myopia on ocular parameters, the understanding of the associated structural changes within the myopic eye remains incomplete. Among these changes, three anatomical domains have been the focus of extensive research: retinal nerve fibre layer (RNFL) thickness, optic nerve head (ONH) morphology, and anterior chamber parameters [9,10,11]. Previous studies [10,12] have identified RNFL thickness as a key structural biomarker of optic nerve integrity. Optical coherence tomography (OCT) findings consistently demonstrate a reduction in peripapillary RNFL thickness with increasing axial length in myopic eyes. This thinning is most pronounced in the superior and inferior quadrants and may be partially offset by increased thickness in the temporal quadrant, probably due to disc tilt and peripapillary atrophy [13-14].

The optic nerve head (ONH) undergoes notable morphological changes in myopia, including increased disc area, disc tilt, and shallower cups due to scleral stretching. In highly myopic eyes, the neuroretinal rim area, which is the area between the disc margin and the optic cup, tends to decrease [15, 16]. Many studies [13-15] have examined RNFL thickness and ONH parameters separately in relation to myopia; few have explored them together or in conjunction with anterior chamber parameters. However, none of these studies investigated the effect of myopia on the RNFL thickness, ONH parameters, and the anterior chamber parameters in one centre of study. Axial elongation in myopic eyes also affects the anterior segment, often leading to an increase in the depth of the anterior chambers and an increase in anterior chamber volumes, which may alter aqueous humour dynamics [11].

Despite advances in imaging techniques such as OCT and Pentacam, few studies have simultaneously assessed RNFL thickness, ONH rim area, and anterior chamber parameters in a cohort of myopic patients. Most normative databases used in imaging devices were based on emmetropic populations, which may not be applicable to moderate or high myopes. There is a notable gap in the literature regarding studies that examine the structural association between RNFL, ONH, and anterior chamber parameters in myopic eyes. Such research is essential for generating evidence-based data and enhancing diagnosis and management, particularly in underrepresented populations like those in Saudi Arabia. Thus, the present study aimed to investigate the association between peripapillary RNFL thickness, ONH rim area, anterior chamber parameters, and axial length in myopic eyes.

## Materials and methods

Study design

This cross-sectional, hospital-based study was conducted at the Royal Commission Medical Centre, Yanbu, Saudi Arabia, between February and May 2025. A total of 152 myopic adults (84 females), aged 18-49 years, were included.

Sample size 

Participants were recruited using a non-probability sampling technique from the optometry and ophthalmology clinics at the Royal Commission Medical Centre. The sample comprised 152 myopic individuals (both male and female) with spherical equivalent refractive errors ranging from -0.75 to -6.75 diopters (D).

Inclusion and exclusion criteria

The inclusion criteria for the study were adults aged 18 to 49 years diagnosed with myopia who agreed to participate and signed an informed consent form. Myopia was defined in this study as a spherical equivalent of refraction (SER) calculated as the sum of the sphere and half the cylinder (½ cyl) ranging from -0.75 to -6.75 D. Whereas exclusion criteria included the presence of astigmatism greater than -0.50 D, amblyopia, strabismus, any ocular pathology that could affect retinal imaging, a history of corneal trauma or corneal refractive surgery, and systemic diseases.

Ethical approval

Ethical approval for conducting the study was obtained from the Medical Ethics and Research Committee of the Royal Commission Health Services Program (approval number RCYMC-EA-2023-01), and the study was conducted in accordance with the principles of the Declaration of Helsinki. Written informed consent was obtained after explaining the study's purpose. Participation was voluntary, with no compensation, and data were collected confidentially without personal identifiers.

Clinical examinations 

Each subject underwent a thorough history-taking process and a comprehensive eye examination to ensure eligibility based on the inclusion criteria. Visual acuity (VA) was assessed using a Snellen chart placed at a distance of 6 metres. Non-cycloplegic refraction was then measured using an autorefractometer (ARK-510A, NIDEK Corporation, Tokyo, Japan) to determine the refractive status. Anterior eye segment examination was performed using slit-lamp biomicroscopy, while the posterior segment was assessed with direct ophthalmoscopy and 90D fundus biomicroscopy.

After detailed eye examinations, eligible subjects underwent full measurements using the Pentacam AXL (OCULUS Optikgeräte GmbH, Wetzlar, Germany). The parameters assessed included central corneal thickness, corneal thickness at the thinnest point, anterior and posterior corneal power, anterior chamber parameters, and axial length. The Pentacam AXL is a modern and widely familiar device for measuring and analysing the anterior segment of the eye. It represents an advanced version of the Pentacam HR, offering enhanced capabilities. In addition, the device provides high-resolution anterior segment tomography; the Pentacam AXL integrates axial length measurement, allowing for a more comprehensive ocular assessment [17]. An important feature of the Pentacam AXL is its ability to assess scan quality using a quality specification (QS) indicator. Scans that meet the required standards are labelled as ‘OK’ in the report. To ensure optimal image quality in the present study, all scans were conducted in a dark room.

Additionally, swept-source OCT (SS-OCT) was used to measure peripapillary RNFL thickness and ONH rim area. These scans were performed using the deep range imaging (DRI) Triton SS-OCT Plus device (Topcon Corporation, Tokyo, Japan), which provides high-resolution imaging of the posterior segment. SS-OCT operates at a scanning speed of 100,000 A-scans per second and uses a wavelength of 1,050 nm. This long wavelength penetrates into deeper tissue than spectral-domain OCT, allowing for detailed imaging of ocular structures such as the retina, choroid, and sclera. Image quality was evaluated on a scale from 0 to 100, with a minimum acceptable score set at 60 to ensure reliable data acquisition. In this study, SS-OCT was used to scan the circumpapillary RNFL using the Optic Disc Cube 200 × 200 protocol. The scan area covered 6 mm × 6 mm around the optic disc, with the scan centred on the optic disc. From each scan, measurements of peripapillary RNFL thickness and ONH rim area were obtained.

Statistical analysis

Data were entered into Microsoft Excel (Microsoft Corporation, Redmond, WA) and analysed using IBM SPSS Statistics software, version 25.0 (IBM Corp., Armonk, NY). Descriptive statistics, including means and standard deviations, were computed for all measured parameters. The Kolmogorov-Smirnov (K-S) test was conducted to evaluate the normality of the data distribution. Bivariate correlation analysis was performed to assess the associations between central corneal thickness, thinnest corneal thickness, peripapillary RNFL thickness, ONH rim area, anterior chamber parameters, and the degree of myopia. A p-value of < 0.05 was considered statistically significant.

## Results

Demographic characteristics of the participants 

The baseline demographic and clinical characteristics of the participants are presented in Table 1. Overall, 152 myopic adults were enrolled, with a mean age of 26.72 ± 9.35 years, more than half of whom were female (55.3%). The one-sample Kolmogorov-Smirnov test confirmed that the distributions of central corneal thickness, thinnest corneal thickness, mean anterior corneal power, RNFL thickness, and anterior chamber parameters were normally distributed, as indicated by p-values greater than 0.05. The mean spherical equivalent for myopia was −2.02 ± 1.34 D. The mean central corneal thickness was 557.78 ± 34.27 μm, while the thinnest corneal thickness was 552.76 ± 33.89 μm. The anterior chamber parameters were as follows: mean angle of 39.77 ± 5.52 degrees, volume of 169.55 ± 32.65 mm³, and depth of 3.10 ± 0.32 mm. The mean peripapillary RNFL thickness was 104.76 ± 9.51 μm, and the mean ONH rim area was 1.28 ± 0.32 mm².

**Table 1:** Summary of descriptive statistics for study variables RNFL: retinal nerve fibre layer

N =152	Minimum	Maximum	Mean	Standard Deviation
Age (years)	18	49	26.72	9.35
Refraction (D)	-0.75	-6.75	-2.02	1.34
Central corneal thickness(μm)	480	656	557.78	34.27
Corneal thickness at thinnest area (μm)	475	654	552.76	33.89
Mean anterior corneal power (dioptre)	32.9	46.95	43.45	1.75
Mean posterior corneal power (dioptre)	-6.9	-5.8	-6.3125	0.23
Anterior chamber angle (degree)	23.4	57.3	39.77	5.52
Anterior chamber volume (mm^3^)	85	247	169.55	32.65
Anterior chamber depth (mm)	2.17	3.91	3.1	0.32
Axial length (mm)	22.29	25.99	23.95	0.67
Peripapillary RNFL thickness (μm)	73	130	104.76	9.51
Optic nerve head rim area (mm²)	0.68	2.44	1.28	0.32

Correlation between peripapillary RNFL thickness, ONH rim area, anterior chamber parameters, and severity of myopia 

**Figure 1:**
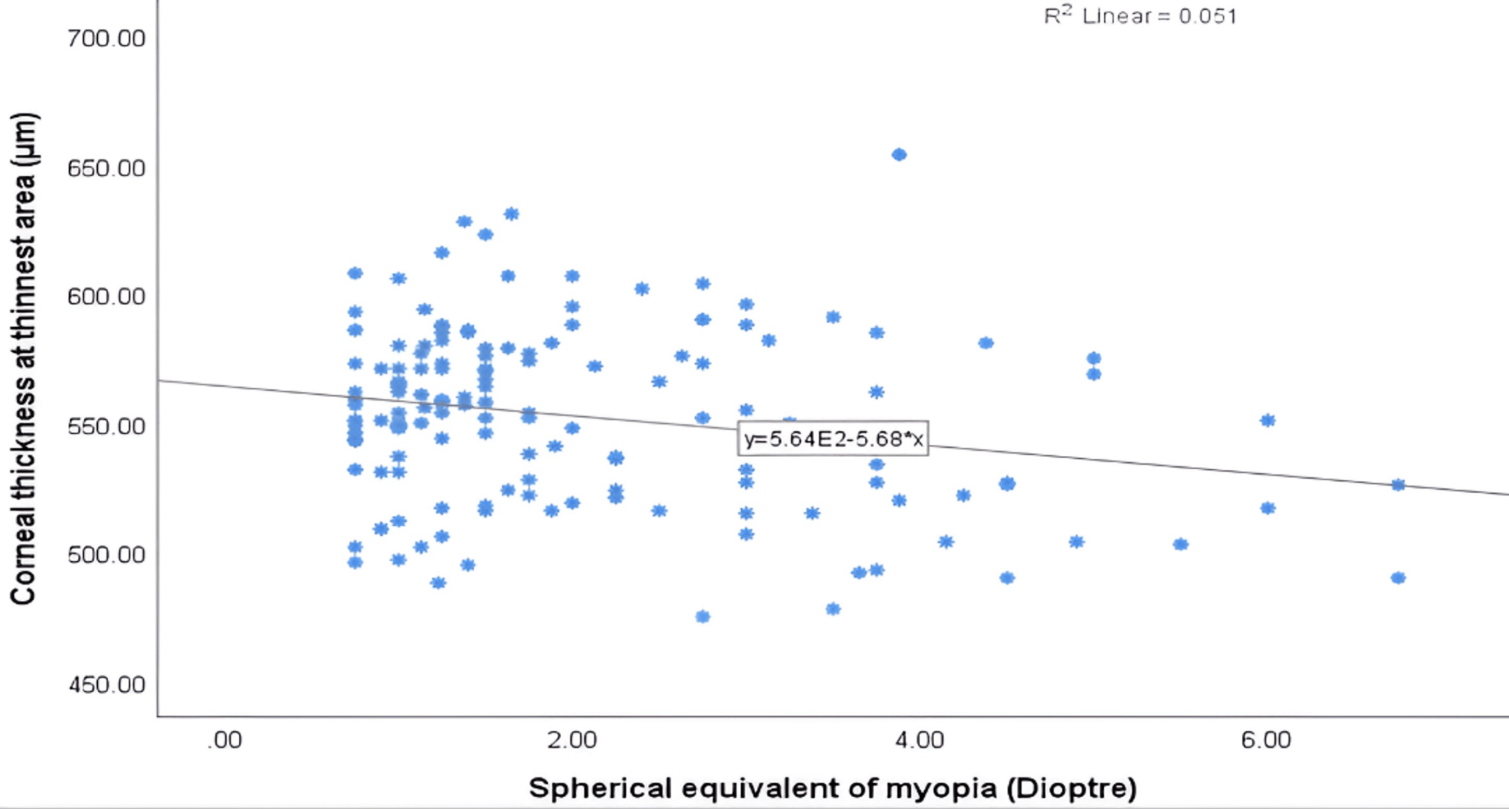
Scatterplot showing the correlation between corneal thickness at the thinnest area and spherical equivalent of myopia.

The correlation coefficients between myopia severity and ocular parameters are presented in Table 2. The findings revealed an inverse correlation between central corneal thickness (r = -0.193*) and corneal thickness at the thinnest area (r = -0.225**) with the severity of myopia, both of which were statistically significant (P < 0.05). This negative correlation between the thinnest corneal thickness and myopia severity is illustrated in Figure 1. There was a weak positive correlation between anterior and posterior corneal power and the degree of myopia, which was not statistically significant (P > 0.05).

**Table 2:** Correlation coefficients between peripapillary Retinal nerve fibre layer thickness, optic nerve head rim area, anterior chamber parameters and degree of myopia (n=152) **: p<0.05

	Myopia (Dioptre) (−2.02 ± 1.34)		
Characteristics	Mean ± S.D	Correlation	P-value
Central corneal thickness (μm)	557.78 ± 34.27	-0.193^*^	0.017
Corneal thickness at thinnest area (μm)	552.76 ± 33.89	-0.225^**^	0.005
Mean anterior corneal power (dioptre)	43.45±1.75	0.088	0.279
Mean posterior corneal power (dioptre)	-6.31± 0.23	0.001	0.994
Anterior chamber angle (degree)	39.77±5.52	0.033	0.685
Anterior chamber volume (mm^3^)	169.55±1.34	0.266^**^	0.001
Anterior chamber depth (mm)	3.10±0.32	0.259^**^	0.001
Axial length (mm)	23.95±0.67	0.545^**^	0
Retinal nerve fibre layer thickness (μm)	104.76±9.51	-0.1	0.222
Optic nerve head rim area (mm²)	1.28±0.32	-0.134	0.101

**Figure 2:**
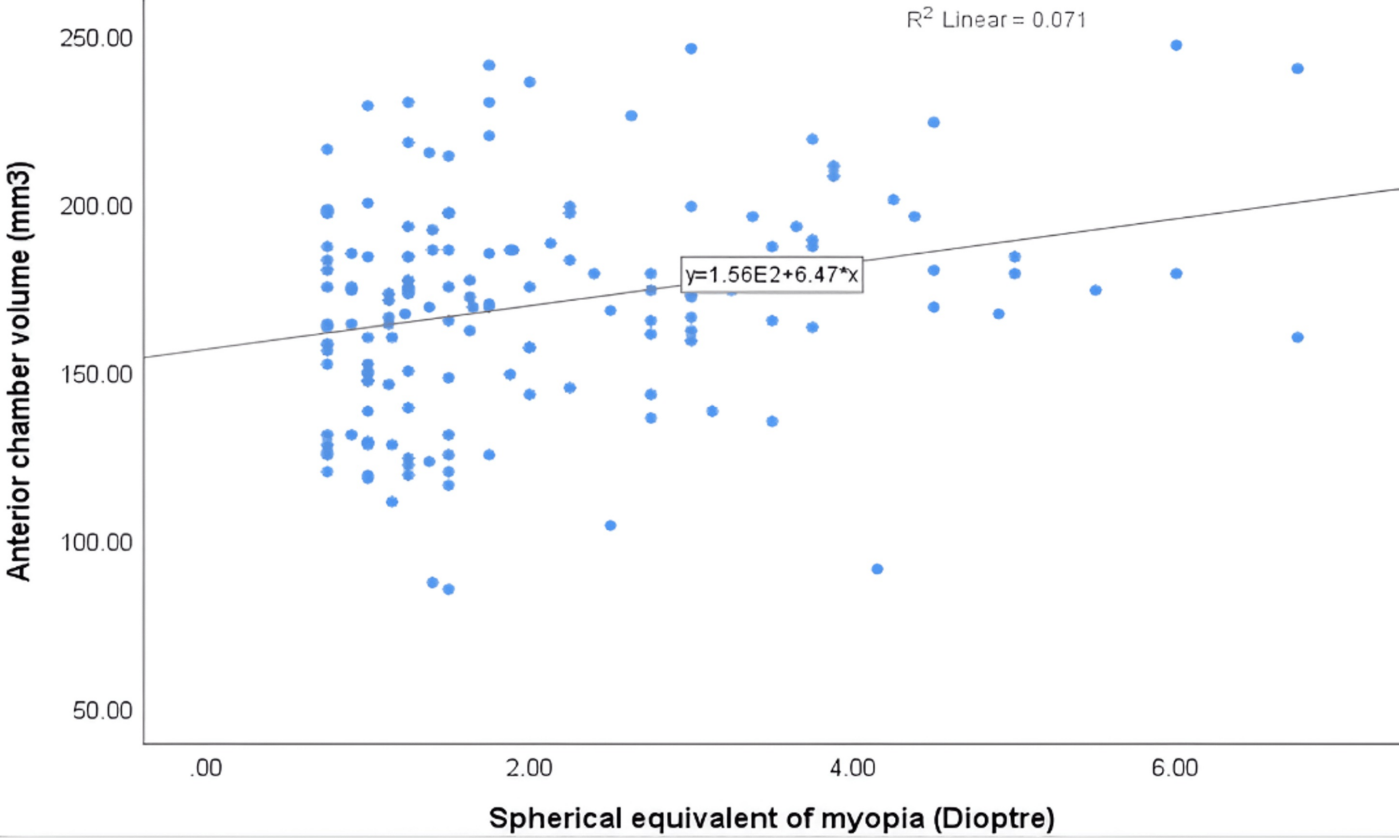
A scatterplot displaying the association between anterior chamber volume and spherical equivalent of myopia.

**Figure 3:**
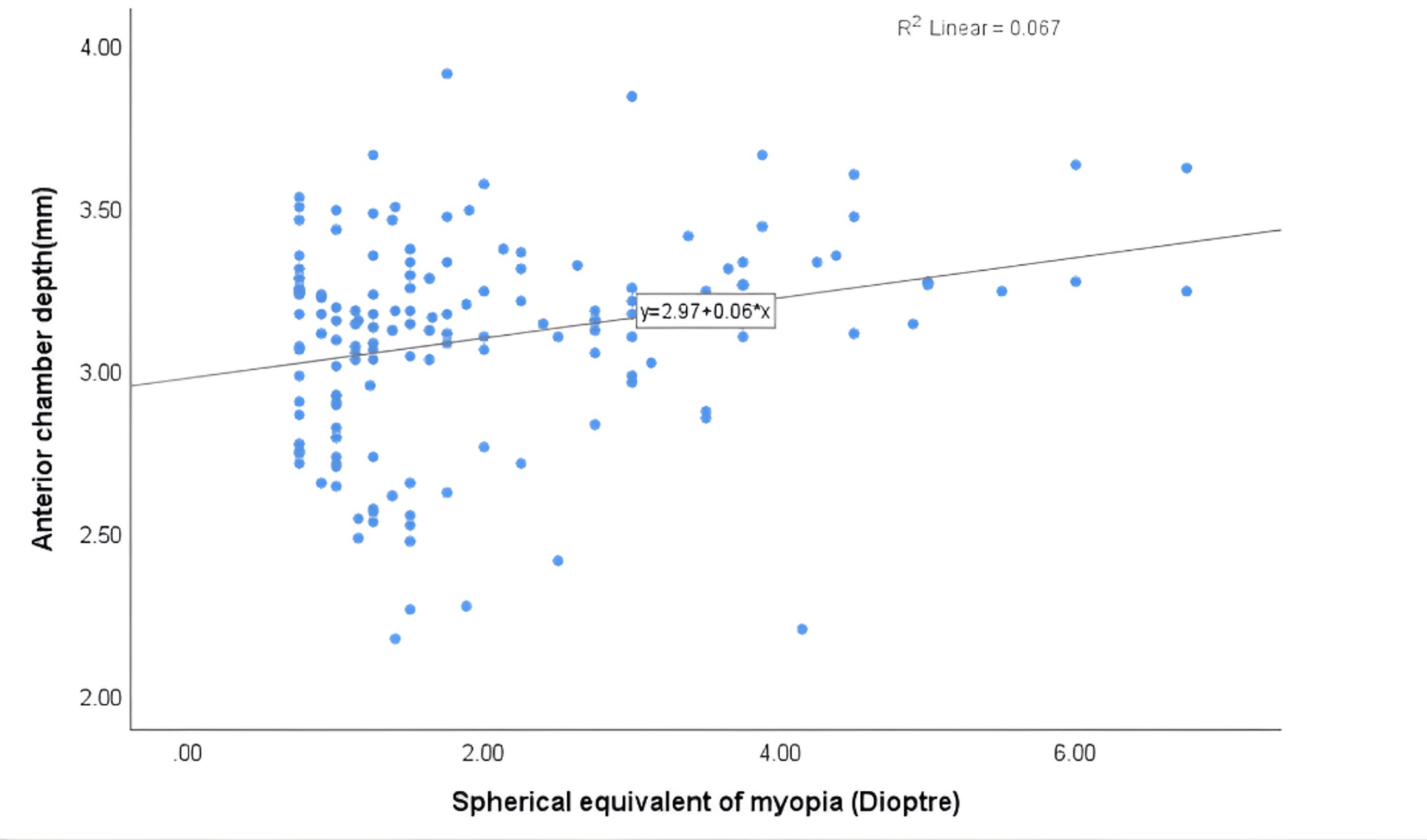
A scatterplot displaying the association between anterior chamber depth and spherical equivalent of myopia.

Regarding anterior chamber parameters, there was a statistically significant positive correlation between anterior chamber volume (r = 0.266**, P < 0.05) and the severity of myopia. The positive association between anterior chamber volume and myopia severity is shown in Figure 2. Similarly, anterior chamber depth showed a significant positive correlation with myopia severity (r = 0.259**, P < 0.05). As shown in Figure 3, anterior chamber depth increased with myopia severity. In contrast, no significant correlation was found between anterior chamber angle and myopia severity (P > 0.05). There was a statistically significant positive correlation between axial length (r = 0.545**) and myopia severity (P < 0.05). There was a weak inverse correlation between peripapillary RNFL thickness and myopia severity (r = -0.100), which was not statistically significant (P > 0.05). Similarly, an inverse correlation was observed between ONH rim area and myopia severity (r = -0.134), but this was also not statistically significant (P > 0.05).

Association between peripapillary RNFL thickness, ONH rim area, anterior chamber parameters, and axial length 

**Figure 4:**
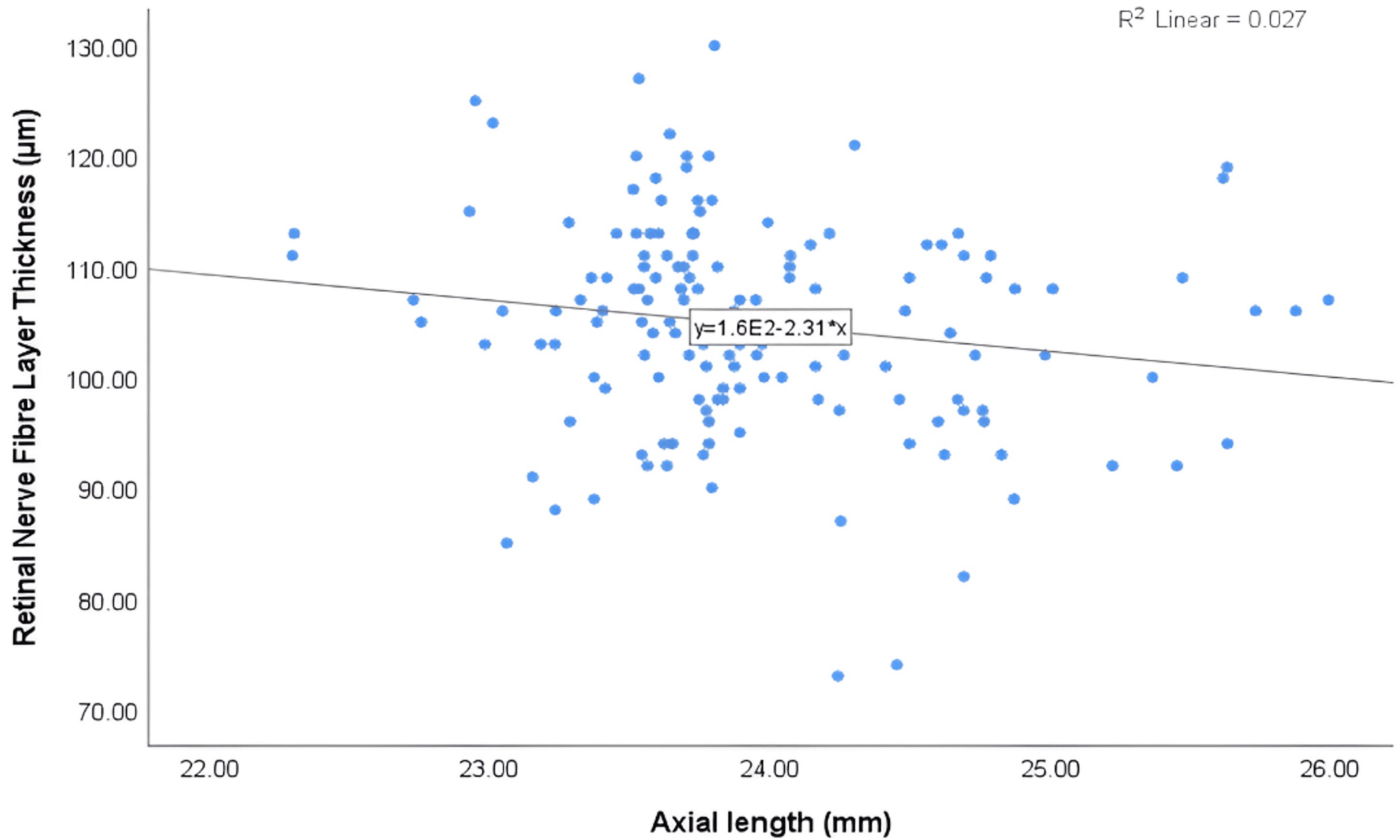
A scatterplot representing the association between retinal nerve fibre layer thickness and axial length.

**Figure 5:**
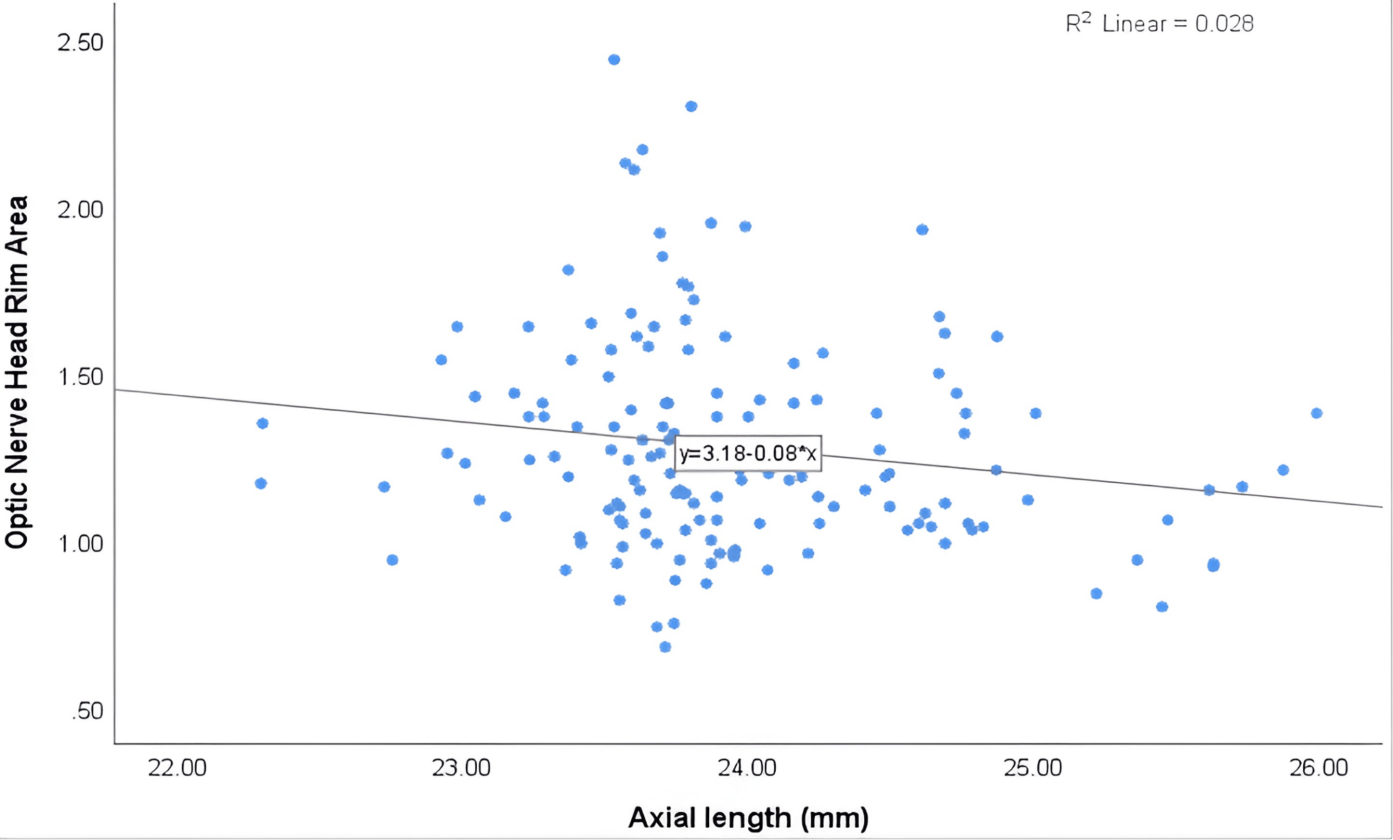
A scatterplot representing the association between optic nerve head rim area and axial length.

The detailed correlations between axial length and ocular parameters are summarised in Table 3. The findings showed a weak inverse correlation between central corneal thickness (r = -0.135) and corneal thickness at the thinnest point (r = -0.150) with axial length. However, neither correlation was statistically significant (P > 0.05). Regarding anterior chamber parameters, there was a statistically significant positive correlation between anterior chamber volume and axial length (r = 0.314**, P = 0.000). Similarly, anterior chamber depth also showed a significant positive correlation with axial length (r = 0.332**, P = 0.000). While no significant association was found between anterior chamber angle and axial length (P = 0.351). Interestingly, there was a statistically significant inverse correlation between peripapillary RNFL thickness and axial length (r = -0.163*, P = 0.044). Figure 4 illustrates a significant inverse correlation between RNFL thickness and axial length. Similarly, an inverse correlation was observed between the ONH rim area and axial length (r = -0.167*, P = 0.040), which was also statistically significant. The inverse association between ONH rim area and axial length is further illustrated in Figure 5.

**Table 3:** Correlation coefficients between peripapillary retinal nerve fibre layer (RNFL) thickness, optic nerve head rim area, anterior chamber parameters and axial length (n=152) *: p<0.05

	Axial length (mm) (23.95±0.67)		
Characteristics	Mean ± S.D	Correlation	P-value
Central corneal thickness (μm)	557.78 ± 34.27	-0.135	0.098
Corneal thickness at thinnest area (μm)	552.76 ± 33.89	-0.15	0.065
Anterior chamber angle (degree)	39.77±5.52	0.076	0.351
Anterior chamber volume(mm^3^)	169.55±1.34	0.314^**^	0
Anterior chamber depth mm)	3.10±0.32	0.332^**^	0
Peripapillary RNFL thickness (μm)	104.76±9.51	-0.163^*^	0.044
Optic nerve head rim area (mm²)	1.28±0.32	-0.167^*^	0.04

Correlation coefficients between age and peripapillary RNFL thickness, ONH rim area, and anterior chamber parameters

Age-related changes in ocular parameters are shown in Table 4. The findings revealed a statistically significant inverse association between age and both central corneal thickness (r = -0.209) and corneal thickness at the thinnest point (r = -0.190*) (P < 0.05). Regarding anterior chamber parameters, a statistically significant negative correlation was observed between anterior chamber volume and age (r = -0.263, P = 0.001). Similarly, anterior chamber depth showed a significant negative correlation with age (r = -0.278**, P = 0.001). Interestingly, the anterior chamber angle also demonstrated a significant inverse association with age (r = -0.197*, P = 0.015). There was a weak, non-significant positive correlation between peripapillary RNFL thickness and age (r = 0.124, P = 0.128). Conversely, a statistically significant inverse correlation was observed between ONH rim area and age (r = -0.198, P = 0.014).

**Table 4:** Correlation coefficients between peripapillary retinal nerve fibre layer (RNFL) thickness, optic nerve head rim area, anterior chamber parameters, axial length and age (n=152) *: p<0.05

	Age (Years) (26.72±9.35)		
Characteristics	Mean ± S.D	Correlation	P-value
Central corneal thickness (μm)	557.78 ± 34.27	-0.209^**^	0.01
Corneal thickness at thinnest area (μm)	552.76 ± 33.89	-0.190^*^	0.019
Anterior chamber angle (degree)	39.77±5.52	-0.197*	0.015
Anterior chamber volume (mm^3^)	169.55±1.34	-0.263^**^	0.001
Anterior chamber depth (mm)	3.10±0.32	-0.278^**^	0.001
Axial length (mm)	23.95±0.67	0.158	0.052
Peripapillary RNFL thickness (μm)	104.76±9.51	0.124	0.128
Optic nerve head rim area (mm²)	1.28±0.32	-0.198^*^	0.014

## Discussion

In myopic eyes, distinct anatomical changes affect both the posterior and anterior segments. These alterations include axial elongation, scleral widening, and enlargement of the lamina cribrosa, all of which may influence optic disc parameters. These structural changes can also extend to the anterior eye segment, potentially modifying corneal curvature and thickness, as well as anterior chamber characteristics such as depth, volume, and angle. These modifications reflect the broader impact of axial elongation on the overall structure and function of the eye. Thus, the current study aimed to investigate the correlation between peripapillary RNFL thickness, ONH rim area, anterior chamber depth, anterior chamber angle, and anterior chamber volume in individuals with myopia. This study highlights significant structural changes in myopic eyes, particularly in relation to corneal thickness, anterior chamber parameters, and optic nerve morphology. 

The observed inverse correlation between myopia severity and both the central and thinnest points of corneal thickness aligns with previous studies suggesting that corneal thinning may be a biomechanical consequence of axial elongation in myopic eyes [11,18]. A recent study has shown a negative correlation between central corneal thickness and both the rate of myopia progression and axial length elongation. This suggests that a thinner cornea may be associated with a fast progression of myopia, highlighting the potential role of corneal biomechanics in the development and progression of refractive errors [19].

The positive associations between anterior chamber volume and anterior chamber depth with the severity of myopia indicate a pattern of anterior eye segment alteration that is consistently observed in myopic eyes. This finding is consistent with a previous study, which showed that anterior chamber depth increases with the severity of myopia [20]. Furthermore, the positive correlation observed between anterior chamber depth and axial length in the present study suggests that deeper anterior chamber depth may be associated with greater axial elongation, potentially reflecting structural adaptations in the myopic eye. These results support the hypothesis which stated that biometric parameters, particularly axial length and anterior chamber depth, play a crucial role in the development and monitoring of myopia. Although our study reinforces existing evidence, further longitudinal research is needed to clarify the causal associations and explore the clinical implications of these associations in different age groups and degrees of myopia [21]. Moreover, an earlier study [11] showed that myopic eyes typically have a deeper anterior chamber depth compared to hyperopic eyes, reflecting structural differences associated with refractive status. The lack of significant association between anterior chamber angle and myopia suggests that not all anterior segment parameters are equally affected by refractive status.

Axial length demonstrated a strong inverse correlation with both peripapillary RNFL thickness and ONH rim area. These findings are consistent with a recent study indicating that axial elongation significantly influences OCT measurements, often leading to reduced RNFL thickness and ONH parameters in myopic eyes [22]. Such structural changes can make glaucoma diagnosis more challenging, as the anatomical alterations seen in high myopia may resemble or conceal true glaucomatous damage [23]. Cakir et al. reported that while RNFL thickness was helpful in diagnosing glaucoma in myopic patients, ONH rim thickness showed superior diagnostic accuracy. This highlights the potential of ORH rim measurements as more reliable indicators of glaucomatous changes in eyes affected by axial elongation [24]. Myopic eyes have been shown to exhibit distinct structural characteristics, including larger optic discs and ONH areas, tilted optic discs, and increased peripapillary atrophy [25,26]. Given the diagnostic challenges posed by high myopia, particularly in differentiating glaucomatous damage from myopia-related anatomical changes, careful interpretation of OCT parameters is essential. Recent evidence highlights the greater diagnostic value of RNFL and ganglion cell-inner plexiform layer thickness compared to ONH metrics in detecting glaucoma among myopic patients [22]. These insights underscore the importance of integrating axial length and macular parameters into routine clinical assessments to enhance diagnostic accuracy and facilitate early detection of glaucoma in myopic populations.

It should also be noted that OCT-derived measurements in highly myopic eyes can be influenced by ocular magnification related to increased axial length. This optical effect may lead to an apparent reduction in peripapillary RNFL thickness, even without true neural loss, and therefore represents an important confounder when interpreting structural OCT findings in myopic patients. Previous research has shown that applying magnification-correction methods, such as those described by Littmann and Bennett, can help minimise this effect and enhance the accuracy of RNFL assessment in eyes with longer axial lengths [12, 13, 23]

 In the present study, the analysis revealed age-related changes in various ocular parameters. A statistically significant negative correlation was found between age and anterior chamber volume (r = -0.263, P = 0.001), as well as anterior chamber depth (r = -0.278, P = 0.001), indicating a reduction in these dimensions with increasing age. Additionally, the anterior chamber angle showed a significant inverse association with age (r = -0.197, P = 0.015), suggesting narrowing of the angle with increasing age. However, a significant inverse association was observed between optic nerve head rim area and age (r = -0.198, P = 0.014), indicating a potential reduction in neuroretinal rim tissue with aging. These findings highlight the importance of considering age-related anatomical changes when evaluating ocular health and disease risk. Recent studies suggest that normal ageing plays a significant role in RNFL thinning, highlighting the importance of accounting for age-related changes when interpreting OCT results. The study revealed that both baseline RNFL thickness and age significantly influence the rate of RNFL decline. This distinction is crucial for differentiating pathological thinning due to glaucoma from physiological thinning associated with ageing or myopic changes [22].

## Conclusions

The study demonstrates that increased myopia is significantly associated with reduced corneal thickness as well as increased anterior chamber volume and depth, while the anterior chamber angle remains unaffected. Additionally, axial elongation shows a strong inverse correlation with peripapillary RNFL thickness and ONH rim area, emphasising the importance of considering axial length and structural changes when evaluating myopic eyes. These findings underscore the importance of assessing RNFL thickness, neuroretinal rim thickness, anterior chamber, and axial elongation in myopic eyes, particularly in the context of ocular diseases such as glaucoma, myopic macular degeneration, and retinal detachment.
